# USF-1 Is Critical for Maintaining Genome Integrity in Response to UV-Induced DNA Photolesions

**DOI:** 10.1371/journal.pgen.1002470

**Published:** 2012-01-26

**Authors:** Yorann Baron, Sébastien Corre, Nicolas Mouchet, Sophie Vaulont, Sharon Prince, Marie-Dominique Galibert

**Affiliations:** 1 CNRS-UMR6061 Genetic and Development Institute of Rennes, RTO Team, Rennes, France; 2 Université de Rennes 1, UEB, IFR140, Rennes, France; 3 The Proclaim Company, Saint-Gregoire, France; 4 Institut Cochin, Université Paris Descartes, CNRS (UMR 8104), Paris, France; 5 Inserm, U1016, Paris, France; 6 Department of Human Biology, Faculty of Health Sciences, University of Cape Town, Cape Town, South Africa; 7 CHU Rennes, Laboratoire de Génomique Médicale, Rennes, France; University of California San Francisco, United States of America

## Abstract

An important function of all organisms is to ensure that their genetic material remains intact and unaltered through generations. This is an extremely challenging task since the cell's DNA is constantly under assault by endogenous and environmental agents. To protect against this, cells have evolved effective mechanisms to recognize DNA damage, signal its presence, and mediate its repair. While these responses are expected to be highly regulated because they are critical to avoid human diseases, very little is known about the regulation of the expression of genes involved in mediating their effects. The Nucleotide Excision Repair (NER) is the major DNA–repair process involved in the recognition and removal of UV-mediated DNA damage. Here we use a combination of *in vitro* and *in vivo* assays with an intermittent UV-irradiation protocol to investigate the regulation of key players in the DNA–damage recognition step of NER sub-pathways (TCR and GGR). We show an up-regulation in gene expression of *CSA* and *HR23A*, which are involved in TCR and GGR, respectively. Importantly, we show that this occurs through a p53 independent mechanism and that it is coordinated by the stress-responsive transcription factor USF-1. Furthermore, using a mouse model we show that the loss of USF-1 compromises DNA repair, which suggests that USF-1 plays an important role in maintaining genomic stability.

## Author Summary

UV is responsible for DNA damage and genetic alterations of key players of the Nucleotide Excision Repair (NER) machinery promote the development of UV-induced skin cancers. The NER is the major DNA–repair process involved in the recognition and removal of UV-mediated DNA damage. Different factors participating in this DNA repair are essential, and their mutations are associated with severe genetic diseases such as Cockayne Syndrome and Xeroderma Pigmentosum. Here, we show for the first time that the specific regulation of expression in response to UV of two NER factors CSA and HR23A is required to efficiently remove DNA lesions and to maintain genomic stability. We also implicate the USF-1 transcription factor in the regulation of the expression of these factors using *in vitro* and *in vivo* models. This finding is particularly important because UV is the major cause of skin cancers and dramatically compromises patients with highly sensitive genetic diseases.

## Introduction

Maintaining the integrity of the genome through cell generations is critical to ensure accurate cell function and to avoid tumor formation. Cells are continuously challenged by environmental insults and they are equipped with specific and efficient defense machinery to remove any DNA alterations. The importance of these processes is underscored by genetic disorders, such as Bloom, Werner, Cockayne Syndromes and Xeroderma Pigmentosum (XP) that result from their impaired function. Despite an enormous amount of progress in identifying the protein complexes and their detailed function in DNA repair pathways, very little is still known about whether these complexes are regulated at a gene expression level.

The skin is a good model in which to address this question because it is the organ most exposed to environmental stresses. The principal cause of DNA damage in the skin is solar irradiation, which induces cyclobutane pyrimidine dimers (CPD) and 6-4 photoproducts in the epidermal cell layers and which, if not removed, can promote skin cancers. The Nucleotide Excision Repair (NER) is the most versatile DNA repair system and is responsible for specifically and constantly eliminating any distorted DNA lesions, including these dimers [1]–[6]. NER can be divided into at least two sub-pathways, Global Genome Repair (GGR) [4] and Transcription Coupled Repair (TCR) [3], [5], [7]. Which one is triggered depends on where the distorted DNA is localized on the genome. GGR, as its name implies, is responsible for removing DNA lesions across the genome including the non-coding part, silent genes and the non-transcribed strands of active genes. The TCR sub-pathway, on the other hand, is dedicated to repairing only DNA lesions detected during transcription and is responsible for removing bulky DNA lesions from the transcribed strands of active genes [2], [3]. The sequence of events implicated in the GGR and TCR DNA repair pathways include: DNA lesion-recognition (the rate limiting step), DNA-unwinding, excision and repair synthesis and except for the damage recognition step, they share common processes and protein machineries for the remaining events [2]. In the GGR sub-pathway, the XPC-HR23 complex is responsible for the recognition of DNA lesions. The DNA-binding protein, XPC, has a strong affinity for damaged DNA [6], [8], [9]. However, its interaction with the evolutionarily conserved HR23 proteins (homologues of the yeast RAD23) is critical for its function. HR23 increases the physiological stability of XPC and thereby its damage recognition activity [10]. In the TCR sub-pathway, lesion recognition occurs through the arrest of the elongating RNA Pol II (RNAPII) when it encounters DNA damage. This essential step initiates the subsequent recruitment of the repair factors CSA and CSB, which are required for the removal of the lesion [5].

While it is well accepted that the functional activity of proteins responsible for the removal of DNA-lesions are regulated and indeed crucial to ensure an orchestrated cascade of events [6], it is not known whether this involves modulation in gene expression. This study addresses this question by using an intermittent UV-irradiation protocol and investigates the gene expression profile of key players in the NER DNA-damage recognition step. We show that UV-induced DNA photo-lesions initiate a specific program of gene expression with the stress responsive transcription factor Upstream Stimulatory Factor 1 (USF-1) playing a central role [11]–[13]. Using a combination of *in vivo* and *in vitro* assays we demonstrate, in our system, that there is a specific and coordinated regulation of *HR23A*, *HR23B*, *CSA* and *CSB* genes and their protein levels in response to UV-mediated DNA damage. We show that up-regulation of both *HR23A* and *CSA* is driven by a common p53 independent mechanism involving USF-1. Furthermore, we provide novel evidence that while HR23A and HR23B share a similar function in DNA-damage recognition, their temporal expressions are different, which may imply that they function at different times, in response to UV-induced DNA-damage.

Results from this study have important implications for our understanding of the role of gene expression regulation in the DNA-damage repair pathways and reveal a role for USF-1 in DNA-repair and in maintaining genome integrity.

## Results

### *CSA* and *HR23A* gene expression is regulated in response to UV-induced DNA damage

**Figure 1:**
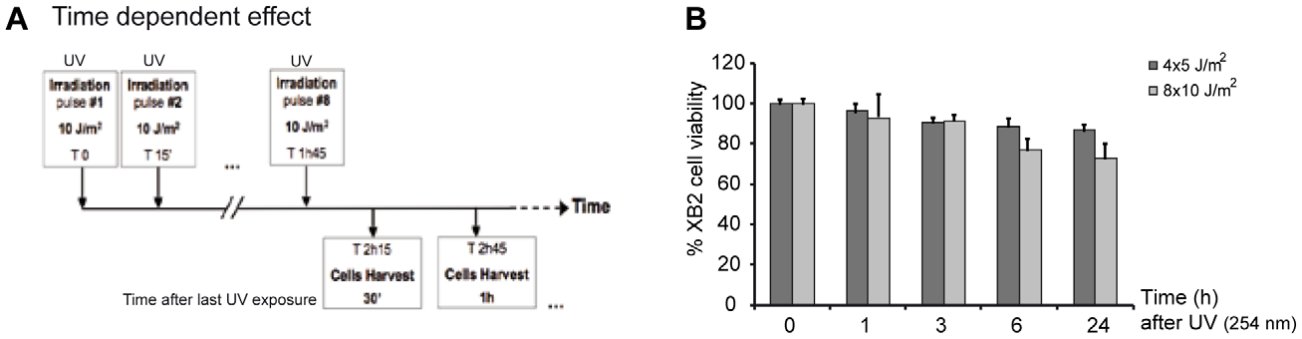
UV-irradiation protocol of XB2 mouse keratinocytes. (A) Schematic representation of the UV (254 nm) treatment time course. Cells were irradiated with eight successive 10 J/m^2^ UV pulses lasting 3 sec each at 15 min intervals, and harvested at indicated times after the last UV pulse. (B) Viability of XB2 keratinocytes after UV-irradiation (4×5 J/m^2^ or 8×10 J/m^2^) was determined by the MTT assay.

**Figure 2:**
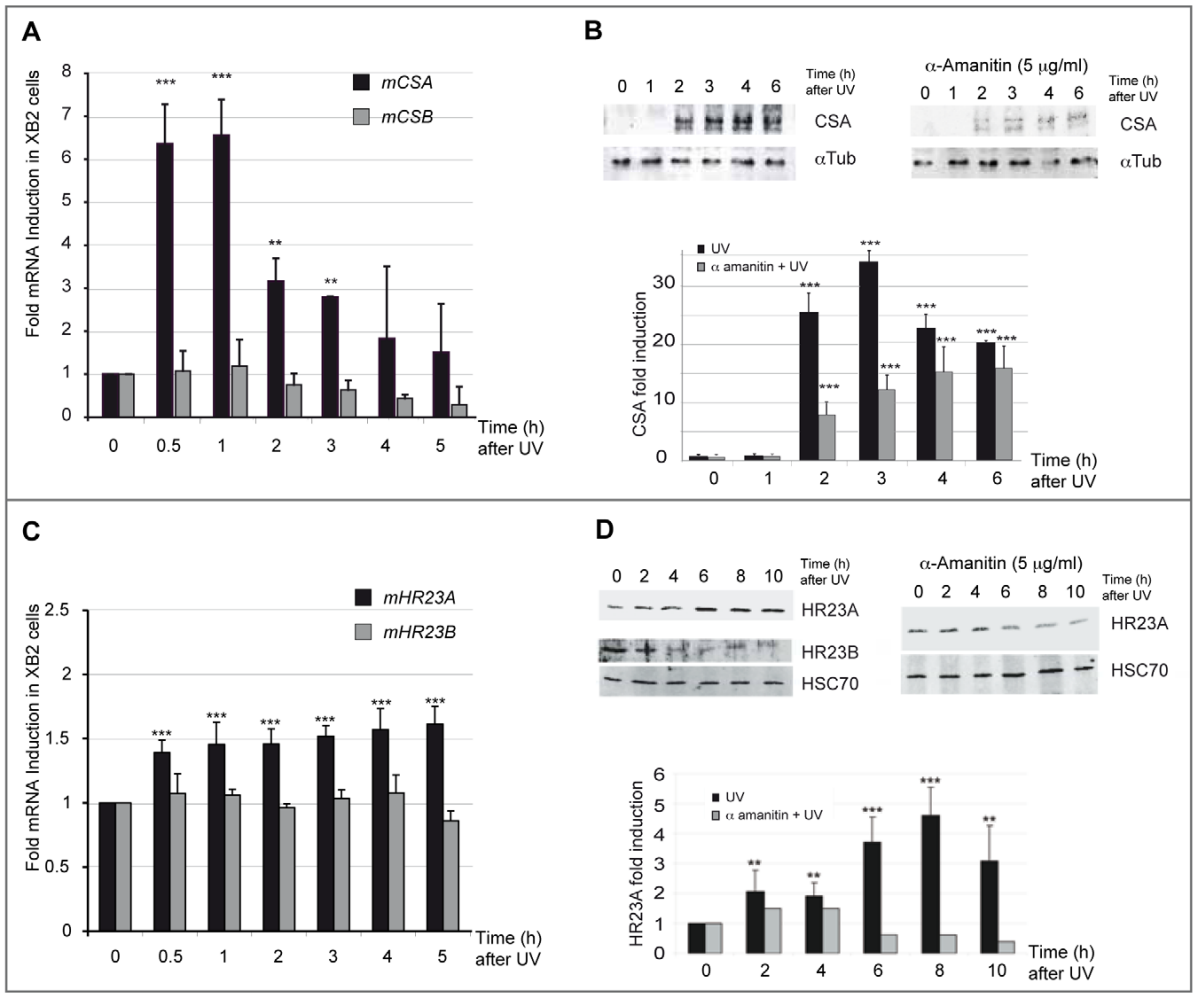
*CSA* and *HR23A* expression is up-regulated in XB2 mouse keratinocytes after repetitive UV irradiation. (A) Quantification of *CSA* and *CSB* expression following UV-irradiation (8×10 J/m^2^) was determined by RT-qPCR (ΔΔCT method). Results are expressed relative to control (no UV treatment) and normalized to an *HPRT* transcript standard (comparable results were obtained with other reference genes: *GAPDH*) n = 3. (B) Western blotting analysis and quantification of CSA protein level in XB2 cells irradiated as previously described and in UV-irradiated cells following a pretreatment or not with α-amanitin. Tubulin (α-Tub) is included as a loading control. Signals are detected using LAS-3000 Imaging System (Fujifilm) and quantified with ImageJ. CSA quantified data are reported in the subpanel, where (▪) corresponds to UV-irradiated samples and (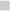) to UV-irradiated samples pre-treated with α-amanitin. The bar graphs compare the intensity of CSA protein normalized to the loading control. (C) Quantification of *HR23A* and *HR23B* mRNA expression following UV-irradiation (8×10 J/m^2^) determined as previously by RT-qPCR (ΔΔCT method) n = 4. (D) Western blotting analysis of HR23A and HR23B protein levels as described previously in irradiated XB2 cells, pre-treated (▪) or not with α-amanitin (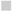). HSC70 is included as a loading control. For all results errors bars indicate s.e.m.; one asterisk, *P*<0,05 *n* = 3; two asterisks, *P*<0.01; three asterisks, *P*<0.001.

Very little is known about how genes that encode key components of the NER recognition step are regulated at a transcriptional level, to mediate their role in DNA lesion recognition. We thus performed a UV-induced DNA-lesion protocol (Figure 1A), which generates immediate DNA photo-lesions through repetitive doses of short wavelength UV pulses rather than delivery of a single high dose [14], [15]. Using RT-qPCR, we then followed the expression of genes specifically involved in the recognition events of TCR (*CSA* and *CSB*) and GGR (*HR23A* and *HR23B*), immediately post-irradiation. Cultured mouse and human keratinocytes (XB2, HaCaT) were irradiated with four to eight 10 J/m^2^ UV pulses (254 nm) at 15 min intervals and collected at the indicated times (from 30 min to 5 h) after the last pulse (Figure 1A). We first checked for the presence of CPD post UV-irradiation (Figure S1) and for cell viability over 24 h confirming that the irradiation procedure was inducing DNA-damage without compromising cell numbers (90% and 75% cell survival at respectively 3 and 24 h) (Figure 1B). The irradiation protocol resulted in a significant increase of *CSA* mRNA levels (6-fold after 30 min), while the abundance of *CSB* gene transcripts was not affected (Figure 2A). *CSA* mRNA levels remained elevated at 1 h and decreased from 2 hours. Comparable results were obtained in p53-deficient human HaCaT keratinocytes [16] (Figure S1A). Up-regulation of *CSA* gene expression was accompanied by a significant increase in CSA protein levels (Figure 2B), peaking at 3 hours compared to un-stimulated cells, where CSA protein is almost undetectable. The increase of CSA protein levels following UV-irradiation was also observed by immunofluoro-staining in XB2 keratinocytes (Figure S1B). This increase in CSA protein levels is significantly reduced over time when cells were pre-treated with α-amanitin, an agent that disrupts transcription. These results indicate that the increase in protein levels results in part from transcriptional regulation.

We next investigated the regulation of the GGR pathway-specific mediators, HR23A and its homologue HR23B, following the same irradiation protocol (Figure 1A). While no significant effect was observed on *HR23B* mRNA levels, the irradiation protocol resulted in a mild but reproducible (6 independent experiments) and significant increase of *HR23A* mRNA levels (1.5-fold at 4 h) (Figure 2C). Comparable results were obtained in p53-deficient human HaCaT keratinocytes (Figure S1C). In parallel with UV-induced *HR23A* transcripts, protein levels increased progressively, reaching a 4-fold increase at 8 hours. This effect was abrogated when cells were pre-treated with α-amanitin (Figure 2D). The increase of HR23A protein levels following UV-irradiation was also observed by immunofluoro-staining in XB2 keratinocytes and correlates with an increase in CPD (Figure S1D). In contrast, HR23B protein levels decreased over time after UV-irradiation suggesting that it is regulated post-transcriptionally since there was no change in its mRNA levels (Figure 2C). These results indicate that HR23A and HR23B are regulated differently.

### UV irradiation promotes the interaction of USF transcription factors with the proximal promoters of *HR23A* and *CSA*

**Figure 3:**
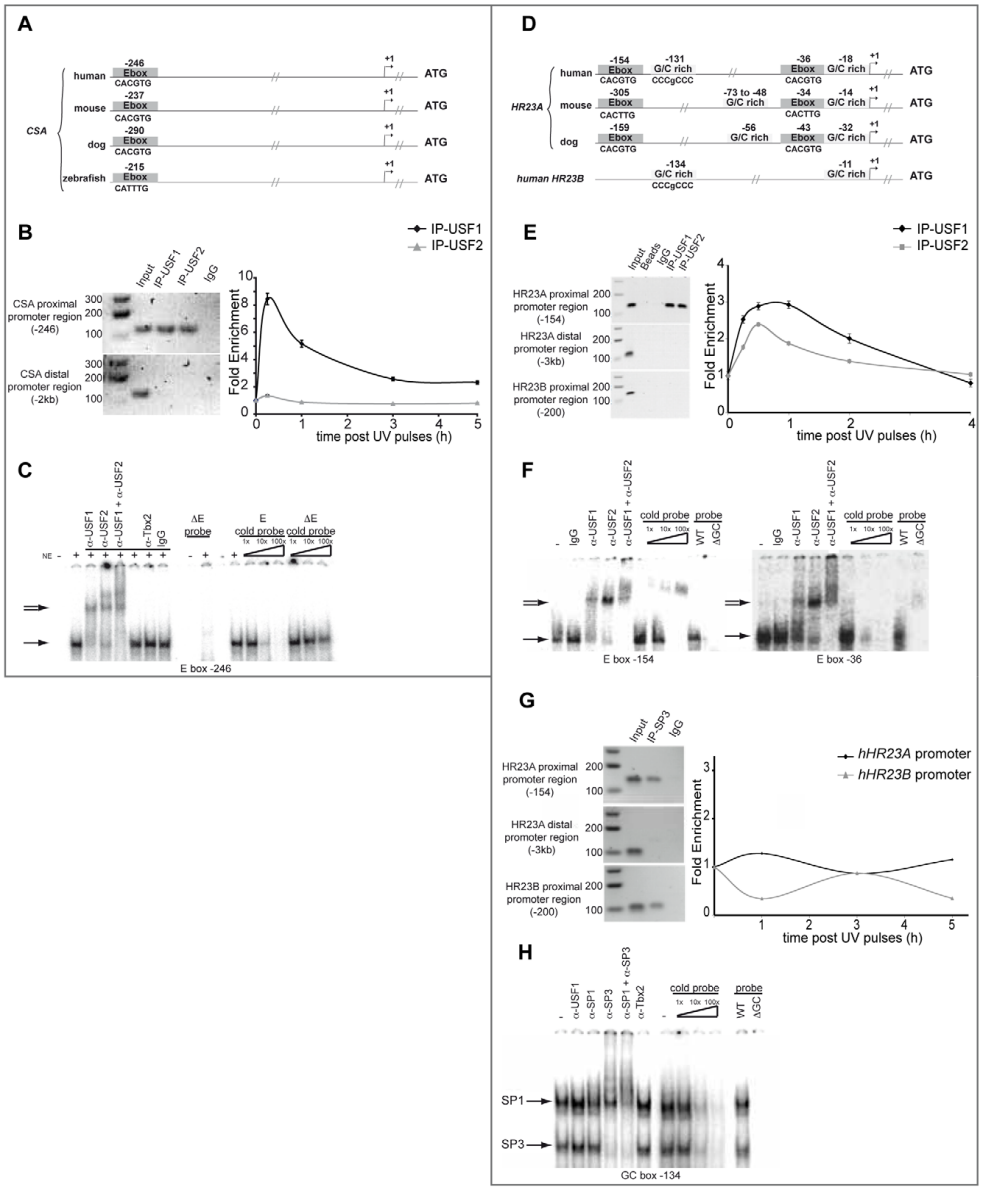
USF family members interact with *CSA* and *HR23A* proximal promoters. (A) Graphic representation of human, mouse, dog and zebrafish *CSA* proximal promoter. Conserved E-boxes are represented in dark grey. (B) *In vivo* chromatin immunoprecipitation assays (ChIP) with HaCaT cells using USF-1, USF-2 antibodies or non-specific IgG. Recovered DNA under basal or UV-irradiation conditions was subjected to PCR or quantitative PCR using specific primers of both proximal and distal region (negative control) of the *CSA* promoter. (C) *In vitro* Electrophoretic Mobility Shift Assay (EMSA) experiments were performed using HaCaT nuclear extract and radiolabeled probes centered on the E-box motif present in the *CSA* proximal promoter (−246) (shifted complex (→)). Competition assays were performed in the presence or not of cold competitors (WT or mutated cold probe). Supershift assays were obtained in the presence of anti-USF-1, anti-USF-2, and anti-TBX2 antibodies or IgG as non-specific controls ( = >). (D) Graphic representation of human, mouse and dog *HR23A* and human *HR23B* proximal promoters. Conserved E-box motifs are represented in dark grey and GC-rich regions in light grey. (E) ChIP assays were performed as in (B) targeting proximal *HR23A* or *HR23B* promoters and the distal region of *HR23A* promoter (−3 kb). (F) EMSA experiments were performed as described in (C) using radiolabeled probes centered on each E-box motif (−154 and −36) present in the *HR23A* proximal promoter. (G) ChIP assay using SP3 antibody or non-specific IgG were performed as previously described for *HR23A* and *HR23B* promoter occupancy. (H) EMSA experiments were performed as previously with HaCaT nuclear extract and radiolabelled probes centered on the GC box present in the *HR23A* proximal promoter (−131) (shifted complex (→)).

UV-induced transcription is a tightly regulated process that involves both *cis* and *trans* UV-responsive elements. We thus explored potential cis/trans factors involved in UV-induced regulation of *CSA* and *HR23A* expression by *in silico* analysis of their respective proximal promoter sequences using Consite and Zpicture (Rvista 2) softwares [17], [18]. We found that both promoters belong to the TATA-less class and that their proximal regions contain consensus E-box motifs (CACGTG) upstream from the transcription start site (TSS) at −246 for *CSA* (Figure 3A) and −154 and −37 for *HR23A* promoter (Figure 3D), which are highly conserved across species. By contrast, no such conserved E-box motif was found in the *CSB* and *HR23B* promoter regions (data not shown and Figure 3D). Given that USF-1 acts as a key player of UV-regulated gene expression by interacting specifically with E-box cis-regulatory elements (CACGTG) as homodimers or as heterodimers with its partner USF-2 [19]–[21], we suspected that *CSA* and *HR23A* may be USF-1 target genes. To test this hypothesis, we performed chromatin immunoprecipitation (ChIP) assays using antibodies specific for either USF-1 or USF-2. DNA recovered from the HaCaT cell line was amplified by PCR using primers targeting distinct promoter sequences (Figure 3B). Results showed specific amplification products corresponding to the binding of USF-1 and USF-2 factors to the *CSA* proximal promoter (−246 bp), whereas no PCR product was observed for the distal region (−2 kb), the proximal *CSB* promoter or with non-specific IgG antibodies (Figure 3B). We next investigated the impact of UV-mediated DNA-damage (8×10 J/m^2^) on the recruitment of USFs to the *CSA* proximal promoter over time. UV-irradiation specifically and rapidly (15 min) promoted an 8-fold enrichment of USF-1, but not USF-2, at the *CSA* proximal promoter (Figure 3B). Using *in vitro* binding assays (EMSA), we tested the ability of USFs to bind the identified conserved E-box motif (−246 bp), which was also present in the ChIP amplified product. Specific DNA-protein complexes were obtained with a probe spanning the E-box motif at −246 (Figure 3C), which were efficiently competed by homologous cold wild type, but not mutant probe. These DNA-protein complexes were super-shifted by antibodies against either USF-1 or USF-2 but not by non-specific antibodies (IgG or Tbx2). No DNA-protein complex was formed with probes carrying mutated E-box sequences.

*In vivo* DNA-binding assays revealed also that USF factors interact specifically with the *HR23A* proximal promoter but not the distal promoter or *HR23B* promoter and that UV-irradiation promotes the interaction of the USF-1 transcription factor by a 3-fold and USF-2 by a 2.5-fold enrichment (Figure 3E). As shown previously for *CSA*, EMSA assays confirmed the DNA-protein complexes spanning the conserved −154 and −36 E-box motifs, present also in the ChIP amplified products (Figure 3F). Competition between the two E-box probes did not reveal any preferential binding site (data not shown). In addition to the E-box sites present in the *HR23A* proximal promoter, *in silico* analysis identified conserved GC-rich regions (−131 and −18 from TSS) (Figure 3D) known to interact with members of the SP1/SP3 transcription factor family [11]. To examine their respective contribution to the regulation of *HR23A* expression, we performed *in vitro* and *in vivo* DNA-binding assays as described above. Specific protein-DNA complexes were formed only in the presence of the −131 intact GC box that interacts with SP1 and SP3 transcription factors (Figure 3G). Also, under the experimental conditions used, only SP3 was able to bind the *HR23A* proximal promoter *in vivo* and SP3 loading was not affected by UV-irradiation. Interestingly, a comparable SP3 binding profile was obtained with the *HR23B* proximal promoter that shares homologous GC rich sequences with *HR23A* (Figure 3H) but whose mRNA levels were not modulated by UV, suggesting that the GC motifs might not be UV-inducible. Taken together these results provided compelling evidence that, in response to UV-irradiation, USF-1 interacts directly with the *CSA* and *HR23A* proximal promoters, suggesting it may be responsible for the UV-induced *CSA* and *HR23A* expression observed in this study.

### USF factors drive *CSA* and *HR23A* gene expression in response to UV via E-box motifs

**Figure 4:**
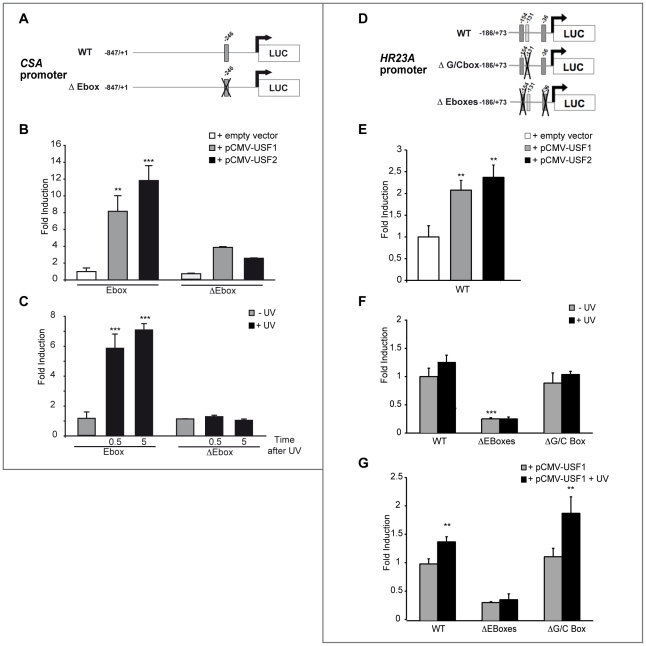
*In vitro* transcriptional regulation of human *CSA* and *HR23A* by USF. (A) Schematic representation of the *CSA* promoter-luciferase constructs. The construct contains the sequence from −847 to +1 of the *CSA* promoter linked to the luciferase reporter. E-box is represented in dark gray and its position is indicated on top. Cross shows mutated E-box. (B) *CSA* promoter-luciferase activity measured after co-transfection of XB2 keratinocytes with WT or mutated CSA promoter-luciferase constructs with pCMV-USF-1, pCMV-USF-2 expression vectors or pCMV empty vector (control). (C) *CSA* promoter-luciferase activity measured 30 min or 5 h after UV induction (6×10 J/m^2^) of XB2 cells transfected with WT or mutated CSA promoter-luciferase constructs. (D) Schematic representation of the *HR23A* promoter-luciferase constructs. The construct contains the sequences from −186 to +73 of the *HR23A* promoter linked to the luciferase reporter. E-boxes are represented in dark gray, GC-box in light gray, and positions are indicated on top. Crosses show mutated boxes. (E) *HR23A* promoter-luciferase activity measured after co-transfection of XB2 keratinocytes with pCMV-USF-1 or pCMV-USF-2 expression vectors or empty vector. (F) WT and mutated *HR23A* promoter-luciferase activities in XB2 cells following UV-irradiation (6×10 J/m^2^). (G) WT and mutated *HR23A* promoter-luciferase activities transfected in XB2 cells with pCMV-USF-1 expression vector and UV irradiated (6×10 J/m^2^). Error bars indicate s.e.m.; *n* = 3; one asterisk, *P*<0.05, two asterisks, *P*<0.01, three asterisks, *P*<0.001.

The relevance of the E-box motifs in mediating USF regulation of the *CSA* and *HR23A* promoters was next assessed by luciferase assays. We first transiently co-transfected XB2 cells with a wild type (WT) and E-box mutated *CSA* promoter (−847/+1) cloned upstream of a luciferase reporter (pGL3-Luc) (Figure 4A) and USF-1 or USF-2 expression vectors (pCMV) [12], [20]. Both USF-1 and USF-2 expression vectors led to significant increases of *CSA*-luciferase activity (Figure 4B). Following UV-irradiation, WT *CSA* promoter activity demonstrated a rapid, 6-fold significant increase (30 min after the last UV pulse) (Figure 4C). Furthermore, this intact E-box *cis*-regulatory element proved to be required for UV-induced activation and to mediate the binding of USF trans-activators (Figure 4B–4C).

We next transiently co-transfected XB2 cells with a WT and E-box mutated *HR23A* promoter (−186/+73) construct cloned upstream of a luciferase reporter (Figure 4D). USF-1 and USF-2 expression vectors led to mild but significant increases of *HR23A*-luciferase activity (Figure 4E). In response to UV-irradiation, *HR23A* promoter activity increased slightly but significantly only in the presence of the USF-1 expressing vector (Figure 4G).

Interestingly, when the two E-box motifs were mutated, we observed a 4-fold reduction of the basal *HR23A*-luciferase activity (Figure 4F) and the USF-1 mediated UV-response was abrogated (Figure 4G). Mutation of the −131 GC-rich motif did not significantly affect *HR23A* basal activity and did not impair the USF-mediated UV-response (Figure 4F–4G), supporting the idea that the UV response is driven by the USF/E-box protein/DNA complexes.

### USF-1 KO mouse tissue shows impaired NER regulation and DNA–damage removal following UV irradiation

**Figure 5:**
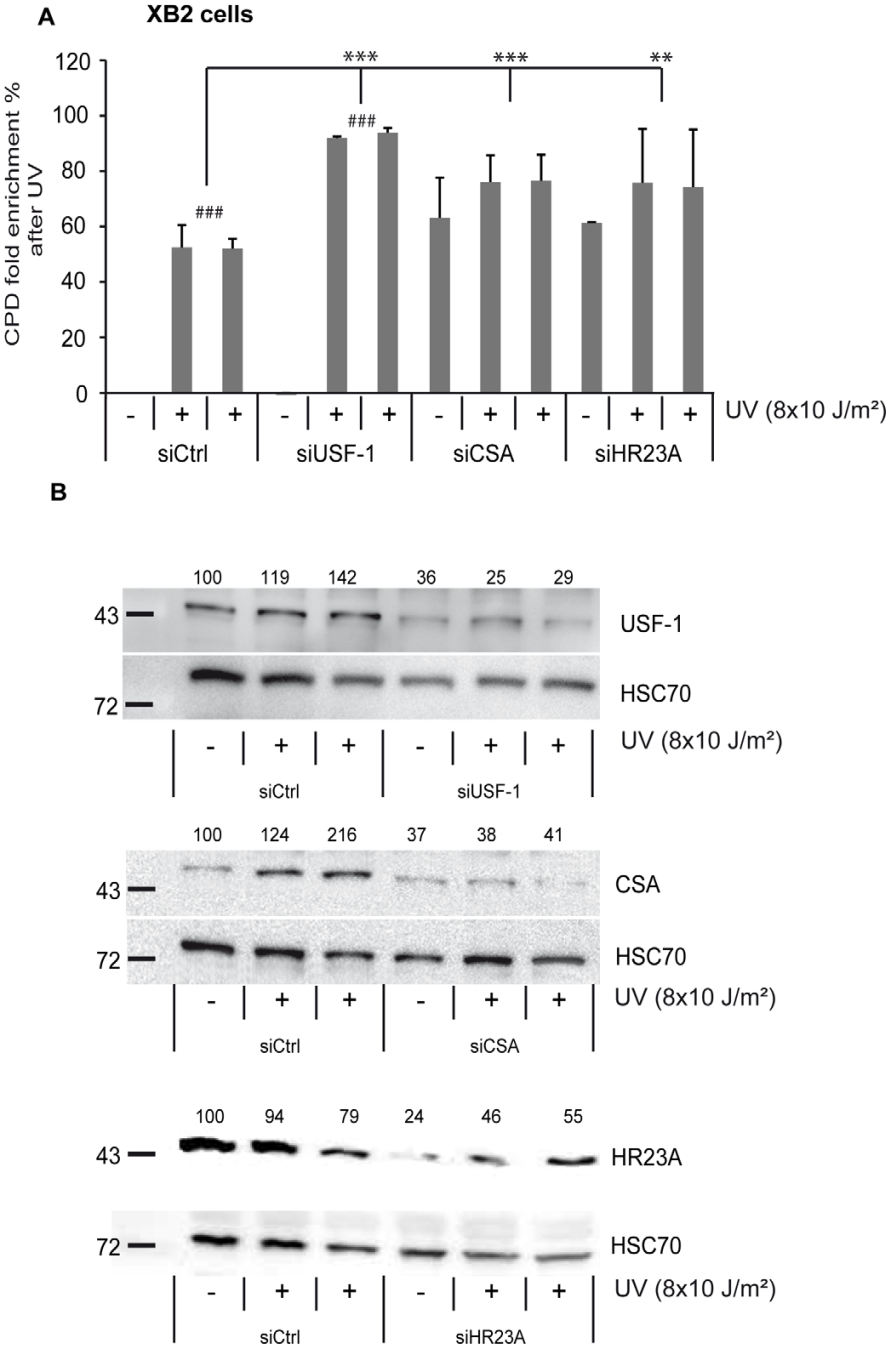
CSA, HR23A, and USF-1 knock-down (KD) affect the level of DNA damage in XB2 cells. (A) ELISA-quantification of CPD DNA-damage in CSA, HR23A and USF-1 KD XB2 cells or control cells, using two independent siRNA (N°1 and 2), 4 hours after UV-irradiation (8×10 J/m^2^). The indicated values correspond to the CPD enrichment (%) against control non UV-irradiated cells. Error bars indicate s.e.m. for three independent experiments. Statistical analysis was performed using student test in order to compare UV conditions and control (#); and between different siRNA target (*) two marks, *P*<0.01, three marks, *P*<0.001. (B) Western blotting analysis and quantification of USF-1, CSA and HR23A protein levels in XB2 cells irradiated as previously described after KD of each target by two independent siRNA (N°1 and 2). HSC70 is included as a loading control. Signals are detected using the LAS-3000 Imaging System (Fujifilm) and quantified with ImageJ.

**Figure 6:**
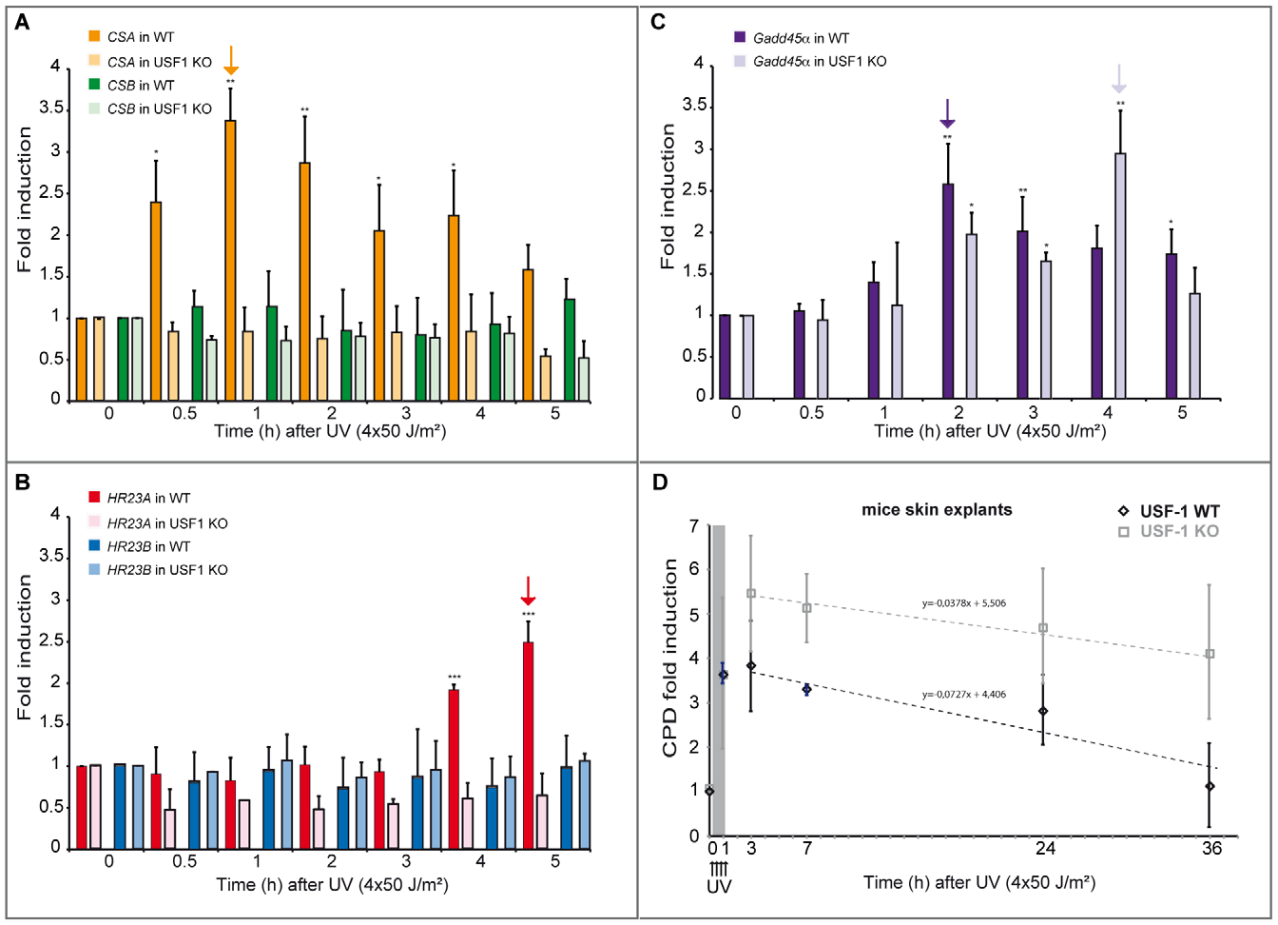
UV-induced *CSA* and *HR23A* expression is impaired in *USF-1* knock-out (KO) mice. (A) Expression analysis of *CSA* and *CSB* were performed by RT-qPCR after UV-irradiation (4×50 J/m^2^) of cultured punch biopsy samples from WT (dark color) or *USF*-1 KO mice (light color). Results for UV-treated samples are expressed relative to controls (no irradiation) with the *HPRT* transcript used as a standard. (B) Expression analysis of *HR23A* and *HR23B* were performed by RT-qPCR as previously described. (C) Expression analysis of the UV response positive control gene, *Gadd45α*, was performed by RT-qPCR as previously described. (D) 36 hours kinetics of CPD DNA-damage removal (ELISA quantification) in cultured skin punch biopsies from WT (black) or *USF-1* KO mice (grey). The yellow band corresponds to the irradiation protocol (4×50 J/m^2^). Skin punch biopsies were analyzed immediately after 3 UV-pulses (3×50 J/m^2^) (time 1 h), and after 4 UV-pulses (4×50 J/m^2^) (at the following times: 3–7–24–36 h). Error bars indicate s.e.m.; *n* = 3.

The physiological significance of the regulation of *CSA* and *HR23A* by USF-1 in response to UV-induced DNA damage was established using genetic approaches with XB2 USF-1 knock-down (KD) cells (Figure 5) and USF-1 knock-out (KO) mice (Figure 6) [13].

Firstly, we quantified the level of CPDs in cells in which either *USF-1* or *CSA* or *HR23A* mRNA was targeted with two different and independent siRNA. While the level of CPDs in the un-stimulated USF-1-KD cells (siUSF-1 N°1 and N°2) remained low and comparable to the control cells (siCtrl N°1 and N°2), the level increased dramatically 4 hours following UV exposure and was significantly higher than the control cells exposed to UV. Surprisingly, although confirmed by two independent siRNAs, levels of CPDs in CSA-KD cells (siCSA N°1 and N°2) and in HR23A-KD cells (siHR23A N°1 and 2) were both significantly elevated in the absence of UV-irradiation compared to USF-1-KD and control cells. Following UV-irradiation, there was a mild increase in levels of CPDs in CSA-KD and HR23A-KD cells which was probably due to the initial high level of CPDs in KD-cells coupled to quantification limits. Nonetheless, these increases remained significantly higher compared to irradiated control cells (Figure 5). Secondly, using skin punch biopsies prepared from USF-1 KO mice and the WT littermates, we analyzed the UV-response by comparing gene transcription efficiency and levels of CPD. RNA analysis comparing irradiated versus non-irradiated WT skin punch biopsies showed that *CSA* and *HR23A* mRNA increased 3.5-fold and 2.5-fold at 1 and 5 h post-irradiation, respectively (Figure 6A–6B). *CSA* and *HR23A* transcript levels remained at basal levels in USF-1 KO mice and *CSB* and *HR23B* mRNA were not affected by UV-irradiation in both WT and KO mice (Figure 6A–6B). By contrast, UV-inducible but USF-1-independent genes, such as the *Gadd45α* prototype displayed UV-induced transcript profiles in WT and KO USF-1 mice (Figure 6C) [22], [23]. However, we detected a 2 h delay of the mRNA increase in the USF-1 KO mice (Figure 6C), which is consistent with RNAPII being arrested to permit DNA-repair of transcribed genes before the commencement of transcription supporting that TCR is compromised in USF-1 KO mice. Moreover, because HR23 proteins are crucial to stabilize XPC at the DNA-photolesion sites to permit removal of damage, we quantified the level of CPD by ELISA immediately after 3 UV-pulses, and after 4 UV-pulses over 36 h (Figure 6D). While basal levels of CPD were comparable in both WT and USF-1 KO mice as expected from siRNA results, UV-irradiation led to rapid increases of DNA-damage that were comparable immediately after 3 UV-pulses but remained higher over time in KO mice compared to WT mice after 4 UV-pulses. Importantly, calculating the rate of CPD-clearance over 36 h, we observed a difference between WT and KO mice (Figure 6D). Whereas CPDs were removed in WT mice at 36 h, CPDs remained elevated in USF-1-KO mice at this time point. Taken together these results provide compelling evidence that in response to UV-induced DNA-damage, loss of USF-1 compromises the tight regulation of the NER resulting in altered removal of UV-induced DNA-damage.

## Discussion

DNA carries the genetic instruction required for the development and functioning of all living organisms. This information must be transmitted to daughter cells with high fidelity, and therefore specific DNA-repair programs are present to eliminate DNA-lesions produced by regular threats. The NER pathway is dedicated to repair distorted DNA, and for decades studies have focused on elucidating the molecular mechanisms involved in the recognition, signaling and removal of these DNA-lesions [2], [24]. Using a multiple dose UV-irradiation protocol with repetitive lower UV-doses that more accurately mimics our exposure to solar irradiation compared to a single high dose, our study identifies an early and coordinated gene expression regulation program of the *CSA* and *HR23A* genes in mammals that relies on the presence of the USF-1 transcription factor.

CSA and CSB proteins have been shown to have dedicated and specific functions in the TCR pathway [5]. It has indeed been clearly established, even in the absence of DNA damage, that a large part of the CSB protein is found associated with chromatin and that RNAPII even in the absence of DNA damage, and this association increases upon UV-irradiation [25], [26]. CSA has however been shown to interact indirectly with RNAPII [25], but it is required in cooperation with CSB for the recruitment of XAB2, HMGN1 and TFIIS, to trigger DNA repair mediated by XP complexes and PCNA protein [5], [24]. The importance of CSA in the early DNA damage response might also reside in the timing of its specific gene expression as its levels are low in resting cells but increase dramatically immediately after UV-irradiation. One possible explanation would be that because CSA acts as a unique player in the initial step of TCR, appropriate levels of the protein is required almost immediately after UV-induced DNA damage and before RNAPII gets arrested by de novo DNA photo-lesions. No increase in CSA protein leads to a delay in transcription likely by an impairment of its associated function: recruitment and stabilization of the initiation complex on the chromatin [25]. This is also supported by deficient CSA being directly linked to the Cockayne syndrome type A genetic disorder [27] and by siRNA results. However, these patients are not prone to developing skin-cancers like XP patients, presumably due to 1- the presence of additional DNA-repair machinery operating post DNA-replication [28], 2- increased cell-death after DNA-damage [28] and to an average life-span for these patients generally being limited to 12 years [29]. Interestingly, specific mutations in the repair-enzyme genes XPB, D and G produce phenotype reflecting a combination of traits present with XP and CS syndromes. This suggests that simultaneous alteration of GGR and TCR will promote mutagenesis in certain cells [29].

HR23A and HR23B proteins share common domains and are both able to form a complex with XPC [30], [31]. The XPC-HR23B complexes were however reported to be more abundant than the XPC-HR23A complexes and have been shown to participate almost exclusively in DNA-photolesions recognition *in vivo* [32]. The XPC-HR23A complexes were consequently regarded as having a functionally redundant role to XPC-HR23B. This is therefore the first study to report conditions under which HR23A and B protein levels are modulated differently, which suggest that HR23A may have a function distinct from HR23B in the UV-induced DNA damage pathway. We show that in response to repetitive UV-irradiation there is a 4-fold increase in the level of HR23A protein which is associated with a concomitant loss of HR23B and we propose that this may favor XPC-HR23A complex formation which leads to sustained XPC-stabilization for appropriate recognition of DNA lesions [32], [33]. Indeed, while HR23A and HR23B KO mice are NER proficient, double HR23A and HR23B KO derived cells show an XPC-like phenotype [34]. We propose that differential regulation of these two HR23 homologues may provide a safety mechanism to ensure the stability of XPC and its function in response to multiple UV-exposure. This possibility is supported by our data that show (i) a reduction of DNA–lesion removal in HR23A-KD cells, (ii) a reduction of DNA-lesion removal in UV-irradiated USF-1 KO tissue and KD cells, which occurs presumably in part due to an abrogation of *HR23A* gene expression in response to UV-rays and (iii) a diminution of HR23A protein when UV-induced *HR23A* transcription is abrogated with α-amanitin. We thus believe that our study reveals a difference in the DNA damage response to a single high dose of UV-irradiation compared to repetitive lower doses and that our conditions mimic the accumulation of DNA-damage over a short period of time which is more applicable to every day life. These results are particularly interesting in the light of the Saccharomyces cerevisiae *RAD23* gene, the ortholog of both *HR23A* and *HR23B*, which also presents with an UV-inducible phenotype [35]. Our results show that the UV-induced function has been conserved through evolution and restricted to one member for specific regulatory purposes.

USF-1 is activated by the stress-dependent p38 kinase and then operates as a transcriptional rheostat of the stress response [20], [21]. Combined regulation of *HR23A* and *CSA* gene expression by USF-1 thus allows a tight and sequential regulation of these two genes. The observation that there is first an increase in USF-1 occupancy on the *CSA* promoter followed by its occupancy on the *HR23A* promoter suggests a sequential and dynamic recruitment of USF-1 to fulfill specific steps of a common task. USF-1 as a stress responsive factor is also proposed to be a key player in regulating pigmentation gene expression in response to UV-irradiation [12], [21], [36]. USF-1 may thus elicit a skin protection program against UV-induced DNA damage by controlling two independent and complementary pathways: the DNA-photolesions repair process and the UV-induced tanning response. More importantly, USF-1 functions independently of p53 but both pathways are expected to be coupled [37]. Since USF-1 mediates an independent and crucial DNA-repair program as highlighted by our USF-1 KO and KD assays, we propose that impairment of this pathway will promote genome instability in response to environmental insults, which is a hallmark of cancer. This hypothesis is supported by the reported loss of USF activity in breast cancer cells [38], and impairment of the recruitment of USF factors to specific E-box elements due to SNPs, as observed in the variant rs1867277 *FOXE1* gene, conferring thyroid cancer susceptibility [39]. Furthermore, CpG methylation can also impair USF interaction with core E-box motifs and subsequently alter gene expression, as for the metallothionein-I gene which is silenced in mouse lymphosarcoma [40].

Our findings indicate that, in response to repetitive environmental threats that lead to the accumulation of UV-induced DNA damage, the NER pathway undergoes a program of gene expression that correlates with the DNA repair processes and that the USF-1 transcription factor is central to this program. These results may thus have important implications for our global understanding of how genome instability is promoted.

## Materials and Methods

### Cell and skin biopsies culture

HaCaT (human - p53 deficient) and XB2 (mouse) keratinocytes were maintained in D-MEM (Invitrogen) supplemented with 10% FBS (Sigma) and 1% Penicillin-Streptomycin (Invitrogen) at 37°C in 5% CO_2_ atmosphere.

Skin biopsies (0.8 cm diameter) were recovered from the backs of WT and USF-1 knockout mice (8 weeks) [13] and maintained in culture for up to 24 h in RPMI (Invitrogen) supplemented with 1% Penicillin-Streptomycin at 37°C in a 5% CO_2_ atmosphere.

### UV irradiation

Specific DNA photo-lesions were generated with ultraviolet bulbs (254 nm) [14], using the Stratalinker apparatus (Stratagene) as previously described [12], [20], [21]. The day before UV exposure, cells were plated at 50–70% confluence, depending on their doubling time, in 10 cm Petri dishes. Twelve to twenty-four later, the medium was replaced with fresh medium supplemented with 2% FBS and 1% antibiotics. The following day, cells were UV irradiated (2× to 8× 10 J/m^2^). UV pulse set at 10 J/m^2^ lasted 3 seconds. The medium was completely removed before and replaced after irradiation. At the time point indicated, cells were washed twice in cold PBS, harvested by scraping, centrifuged and resuspended in appropriate buffer. For transcription inhibition experiments, cells were pre-treated with α-amanitin (5 µg/ml; Sigma) 30 min prior to UV-irradiation.

Mouse skin biopsies were irradiated with four successive pulses of 50 J/m^2^ UV, recovered at the indicated time points by placing the skin biopsy directly in RNA later buffer (Qiagen) and stored at −20°C for subsequent RNA extraction.

### Cell viability test

Cell viability in response to UV (254 nm) was analysed in 96 well plates. Briefly, cells were plated at 1×10^4^ cells/well, 10 h before UV induction, tetrazolium salt (MTT, 0.5 mg/ml (Sigma) was added to culture medium. After 3 h of incubation (37°C), the medium was removed and 150 µl of DMSO was added to each well. Percentage of cell viability was then analysed by measuring the DMSO-optical density (OD), at 690 and 540 nm with a Multiskan spectrophotometer.

### Gene expression analysis

RNA was extracted using Nucleospin RNA II kit (Macherey Nagel) and quantified using the Nanodrop device. For skin explants, an extra Trizol/chloroform purification step was needed to remove protein. cDNA was obtained by reverse transcription using a High-Capacity cDNA Reverse Transcription Kit (Applied Biosystem) from 1 µg of total RNA. Gene expression was analyzed by qPCR in sealed 384-well microtiter plates using the SYBR Green TM PCR Master Mix (Applied Biosystem) with the 7900HT Fast Real-Time PCR System (Applied Biosystem). Relative amounts of transcripts were determined using the delta Ct method. The mRNA levels at each time point following stimulation are expressed as fold increase, relative to non-irradiated cells. Data were normalized independently to at least two housekeeping genes HPRT and GAPDH. Because comparable data were obtained only the HPRT ones are presented. Each experiment was carried out at least twice and each time point was repeated in triplicate. Forward (F) and reverse (R) primers were designed using the Universal Probe Library Assay Design Center (Roche) and have been previously tested for their efficiency (Sequences available on request).

### Western blot analysis

Harvested cells were immediately lysed by incubation for 30 min in ice-cold RIPA buffer (supplied in protease and phosphatase inhibitors). Equal amounts of protein were denaturated in Laemmli buffer for 5 min at 95°C and resolved by 15% SDS-PAGE. Membranes were probed with appropriate antibodies and signals detected using the LAS-3000 Imaging System (Fujifilm) were quantified with ImageJ (http://rsbweb.nih.gov/ij/).

### Electrophoresis mobility shift assay

Gel electrophoresis DNA binding assays were performed with crude HaCaT keratinocyte nuclear extracts under conditions previously described [12], [41], [42], with modifications. Double-stranded oligonucleotides were labeled with T4 polynucleotide kinase in the presence of P^32^-γATP (3000 Ci/mmol) and purified in columns (Mini Quick Spin Oligo Columns, Roche Diagnostic). Reaction mixtures contained 2–4 µg of total protein and 0.03 pmol of P^32^ end-labelled probe in binding buffer (Hepes 25 mM, KCl 150 mM, 10% Glycerol, DTT 10 mM, 1 µg of poly(dIdC), 1 µg salmon sperm DNA). After 20 min of incubation, samples were loaded onto a low ionic strength 6% polyacrylamide gel (29∶1 cross-linking ratio) containing Tris Borate Na EDTA buffer pH 8.3.

Supershift and competition assays were performed by adding competitor probes (1× to 100×) or antibodies (0.2 µg) prior to incubation with labelled probes (Sequences available on request). Radioactive bands were quantified with a STORM 840 PhosphorImager (Molecular Dynamics).

### Chromatin immunoprecipitation assay

ChIP assays, using 1.5–2×10^6^ HaCaT cells, were performed as previously described [41], [43], with specific adaptations. The cells were cross-linked (1.5% formaldehyde), washed twice and collected in 1 ml cold PBS. Cells were lysed and the samples were then sonicated for DNA fragmentation (Sonifier Cell Disruptor, Branson) in 1 ml lysis buffer (10 mM EDTA, 50 mM Tris-HCl (pH 8.0), 1% SDS, 0.5% Empigen BB) and diluted 2.5-fold in IP buffer (2 mM EDTA, 100 mM NaCl, 20 mM Tris-HCl (pH 8.1), 0.5% Triton X-100). This fraction was subjected to immunoprecipitation overnight with 3 µg of the appropriate antibody. These samples were then incubated for 3 h at 4°C with 50 µl of protein A-Sepharose beads slurry. Precipitates were washed several times, cross-linking reversed and DNA purified using a Nucleospin Extract II kit (Macherey Nagel).

PCR or qPCR analyses were carried out with primers spanning *HR23A*, *HR23B* and *CSA* proximal promoters or, as a reference, with primers targeting an unrelated promoter region (HSP70 promoter region) or unspecific regions of target promoter genes (sequences available on request). End-point PCR was performed in semi-quantitative conditions for ChIP (30 amplification Cycles). For qPCR analysis, fold enrichment was determined using the ΔΔCt method: Fold enrichment = 2^−(Δct1−ΔCt2)^, where ΔCt 1 is the ChIP of interest and ΔCt2 the control ChIP.

### Plasmid constructs

−744/+73 and −185/+73 HR23A promoter region were obtained by PCR and inserted into the luciferase reporter plasmid pGL3-basic (Promega). E boxes and the GC box were mutated using a QuickChange Site-Directed Mutagenesis Kit (Stratagene). The same protocol was used for the CSA promoter sequence lying −847/+1.

### Luciferase reporter analysis

*CSA* and *HR23A* promoter regulation was studied in mouse XB2 keratinocytes. Cells were plated at 60–70% confluence in 12-well plates in medium supplemented with 10% SVF without antibiotics and were maintained for 12 h. Cells were co-transfected or not with pGL3 reporter vector and pCMV (empty, USF-1 or USF-2), as previously described [12], [20], [21]. The transfection mix, containing up to 500 ng of plasmid DNA, was prepared in Optimem medium (Invitrogen) and used to transfect cells for 3 h using Lipofectamin 2000 (Invitrogen). 3 h after transfection, the medium was replaced with fresh medium supplemented with 10% SVF and 1% antibiotics. 48 h later, cells were irradiated with UV, as described above and harvested up to 5 h following UV. Cells were then passively lysed and luciferase activity was quantified in a Microplate Luminometer Centro LB 960 (Berthold) using the Luciferase Reporter Assay System (Promega).

### siRNA transfection

XB2 cells were seeded and transfected in 10 cm-diameter dishes (1×10^6^ cells per dish) in DMEM medium complemented with 10% FBS, with 40 pmol of siRNA. Two different siRNA (N°1 and 2) were used independently for each target gene tested (*CSA*, *HR23A* and *USF-1*) as for control (siOTP1, siNT1) (Sigma-Genosys, St Louis, MO) using Lipofectamine 2000 (Invitrogen, Paisley, UK). Transfections were performed following provider's instructions. 72 hours later, the cells were UV irradiated as previously described and recovered 4 hours after the irradiation protocol for CPD quantification and western blot analysis. siRNA sequences are available on request.

### CPD quantification by ELISA

Quantifications of CPD in skin explants following UV (254 nm) (4×50 J/m^2^) were performed by ELISA, accordingly to Cosmo bio recommendations. DNA purification was performed by phenol/chloroform extraction and ethanol precipitation. Briefly, 200 ng of denatured DNA was distributed onto protamine sulfate precoated 96 well plates (Polyvinylchloride flat-bottom). Detection of DNA-lesion was performed using specific mouse anti-CPD antibodies, and revealed with the biotin/peroxidase-streptovidin assay. Quantification was obtained by the absorbance at 492 nm. Each experiment was performed independently with punch biopsies of three independent WT and USF-1 KO mice.

### Immunofluorescence microscopy

XB2 (mouse keratinocytes) cell lines were cultured in D-MEM at 37°C on glass coverslips in 35-mm dishes. 24 hours later, cells were UV-irradiated with 6×10 J/m^2^ in serum free medium following as previously described. Cells were then fixed and permeabilized after different times of induction accordingly to Cosmo bio Co protocol. Previously to CPD immunostaining in cells, we denatured DNA with HCl 2 M for 30 min at room temperature. Indirect immunofluorescence was then performed using specific recommendations of Cosmo bio Co protocol with specific primary antibodies mouse anti-CPD (TDM2 clone, MBL) (1∶3000). Fluoro-staining was performed with labeled donkey anti-mouse IgG (Alexa Fluor 488). CSA immunostaining was performed with specific anti-rabbit antibody from Santa Cruz.

### Antibodies

Anti USF-1 (C:20), USF-2 (N-18), Sp1 (PEP 2), Sp3 (D-20), TBX-2 (C-17), HR23B (P-18), HSC70 (B-6) were purchased from Santa Cruz. Anti HR23A (ARP42211) was purchased from Aviva. Anti CSA was purchased from Abcam (ab96780). Anti CPD (TDM2) was purchased from MBL. Anti α-Tubulin (ARP42211) was purchased from Sigma.

### Statistical analysis

Errors bars represent standard deviation, stars indicate statistically significant differences (two-tailed Student's t-test) between control and irradiated samples * P<0.05; ** P<0.01; *** P<0.001.

### Ethics statement

The present animal study follows the 3R legislation (Replace-Reduce-Refine). It has been declared and approved by the French Government Board. Animal welfare is a constant priority: animals were thus sacrificed under anesthesia.

## Supporting Information

Figure S1 *CSA* and *HR23A* are up-regulated in p53-deficient HaCaT human keratinocytes and XB2 mice keratinocytes after UV induced DNA-damage. (A) Quantification of *CSA* and *CSB* expression in human HaCaT keratinocytes (p53 deficient cells) following UV-irradiation (8×10 J/m^2^) determined by RT-qPCR (ΔΔCT method). Results (n = 3) are expressed relative to control (no UV treatment) and normalized to *HPRT* transcript. (B) Fluoro-immunostaining microscopy (×63) performed in irradiated (6×10 J/m^2^) or not XB2 keratinocyte cells, and recovered 1 and 5 h post-irradiation. Detection of CSA protein was performed using the specific anti-CSA antibody (Santa Cruz) and the secondary TRITC-coupled antibody. DAPI staining was used to visualize cell nuclei. (C) Quantification of *HR23A* and *HR23B* mRNA expression in human HaCaT keratinocytes as described in (A). (D) Fluoro-immunostaining microscopy (×40) performed in XB2 keratinocyte cells, irradiated or not (6×10 J/m^2^), and recovered at 4 h and 24 h post-irradiation. Detection of HR23A protein was performed using the specific anti-HR23A antibody (Aviva) and the secondary TRITC-coupled antibody. Specific anti-CPD antibody (MBL) was used to visualize DNA damage (secondary antibody used was coupled to FITC). (For all results errors bars indicate s.e.m.; *n* = 3; one asterick, *P*<0.05, two asterisks, *P*<0.01, three asterisks, *P*<0.001).
(TIF)

## References

[1] Le May N, Mota-Fernandes D, Velez-Cruz R, Iltis I, Biard D (2010). NER factors are recruited to active promoters and facilitate chromatin modification for transcription in the absence of exogenous genotoxic attack.. Mol Cell.

[2] Nouspikel T (2009). DNA repair in mammalian cells: Nucleotide excision repair: variations on versatility.. Cell Mol Life Sci.

[3] Sarasin A, Stary A (2007). New insights for understanding the transcription-coupled repair pathway.. DNA Repair (Amst).

[4] Sugasawa K (2010). Regulation of damage recognition in mammalian global genomic nucleotide excision repair.. Mutat Res.

[5] Tornaletti S (2009). DNA repair in mammalian cells: Transcription-coupled DNA repair: directing your effort where it's most needed.. Cell Mol Life Sci.

[6] Volker M, Mone MJ, Karmakar P, van Hoffen A, Schul W (2001). Sequential assembly of the nucleotide excision repair factors in vivo.. Mol Cell.

[7] Laine JP, Egly JM (2006). When transcription and repair meet: a complex system.. Trends Genet.

[8] Sugasawa K, Ng JM, Masutani C, Iwai S, van der Spek PJ (1998). Xeroderma pigmentosum group C protein complex is the initiator of global genome nucleotide excision repair.. Mol Cell.

[9] Yokoi M, Masutani C, Maekawa T, Sugasawa K, Ohkuma Y (2000). The xeroderma pigmentosum group C protein complex XPC-HR23B plays an important role in the recruitment of transcription factor IIH to damaged DNA.. J Biol Chem.

[10] Kusumoto R, Masutani C, Sugasawa K, Iwai S, Araki M (2001). Diversity of the damage recognition step in the global genomic nucleotide excision repair in vitro.. Mutat Res.

[11] Al-Sarraj A, Day RM, Thiel G (2005). Specificity of transcriptional regulation by the zinc finger transcription factors Sp1, Sp3, and Egr-1.. J Cell Biochem.

[12] Corre S, Primot A, Sviderskaya E, Bennett DC, Vaulont S (2004). UV-induced expression of key component of the tanning process, the POMC and MC1R genes, is dependent on the p-38-activated upstream stimulating factor-1 (USF-1).. J Biol Chem.

[13] Vallet VS, Casado M, Henrion AA, Bucchini D, Raymondjean M (1998). Differential roles of upstream stimulatory factors 1 and 2 in the transcriptional response of liver genes to glucose.. J Biol Chem.

[14] Perdiz D, Grof P, Mezzina M, Nikaido O, Moustacchi E (2000). Distribution and repair of bipyrimidine photoproducts in solar UV-irradiated mammalian cells. Possible role of Dewar photoproducts in solar mutagenesis.. J Biol Chem.

[15] Pfeifer GP, You YH, Besaratinia A (2005). Mutations induced by ultraviolet light.. Mutat Res.

[16] Lehman TA, Modali R, Boukamp P, Stanek J, Bennett WP (1993). p53 mutations in human immortalized epithelial cell lines.. Carcinogenesis.

[17] Loots GG, Ovcharenko I (2004). rVISTA 2.0: evolutionary analysis of transcription factor binding sites.. Nucleic Acids Res.

[18] Sandelin A, Wasserman WW, Lenhard B (2004). ConSite: web-based prediction of regulatory elements using cross-species comparison.. Nucleic Acids Res.

[19] Corre S, Galibert MD (2005). Upstream stimulating factors: highly versatile stress-responsive transcription factors.. Pigment Cell Res.

[20] Corre S, Primot A, Baron Y, Le Seyec J, Goding C (2009). Target gene specificity of USF-1 is directed via p38-mediated phosphorylation-dependent acetylation.. J Biol Chem.

[21] Galibert MD, Carreira S, Goding CR (2001). The Usf-1 transcription factor is a novel target for the stress-responsive p38 kinase and mediates UV-induced Tyrosinase expression.. Embo J.

[22] Hollander MC, Alamo I, Jackman J, Wang MG, McBride OW (1993). Analysis of the mammalian gadd45 gene and its response to DNA damage.. J Biol Chem.

[23] Mouchet N, Adamski H, Bouvet R, Corre S, Courbebaisse Y (2010). In vivo identification of solar radiation-responsive gene network: role of the p38 stress-dependent kinase.. PLoS ONE.

[24] Fousteri M, Mullenders LH (2008). Transcription-coupled nucleotide excision repair in mammalian cells: molecular mechanisms and biological effects.. Cell Res.

[25] Fousteri M, Vermeulen W, van Zeeland AA, Mullenders LH (2006). Cockayne syndrome A and B proteins differentially regulate recruitment of chromatin remodeling and repair factors to stalled RNA polymerase II in vivo.. Mol Cell.

[26] van Gool AJ, Citterio E, Rademakers S, van Os R, Vermeulen W (1997). The Cockayne syndrome B protein, involved in transcription-coupled DNA repair, resides in an RNA polymerase II-containing complex.. Embo J.

[27] van Hoffen A, Natarajan AT, Mayne LV, van Zeeland AA, Mullenders LH (1993). Deficient repair of the transcribed strand of active genes in Cockayne's syndrome cells.. Nucleic Acids Res.

[28] Hoeijmakers JH (2009). DNA damage, aging, and cancer.. N Engl J Med.

[29] Andressoo JO, Hoeijmakers JH (2005). Transcription-coupled repair and premature ageing.. Mutat Res.

[30] Li L, Lu X, Peterson C, Legerski R (1997). XPC interacts with both HHR23B and HHR23A in vivo.. Mutat Res.

[31] Sugasawa K, Ng JM, Masutani C, Maekawa T, Uchida A (1997). Two human homologs of Rad23 are functionally interchangeable in complex formation and stimulation of XPC repair activity.. Mol Cell Biol.

[32] Okuda Y, Nishi R, Ng JM, Vermeulen W, van der Horst GT (2004). Relative levels of the two mammalian Rad23 homologs determine composition and stability of the xeroderma pigmentosum group C protein complex.. DNA Repair (Amst).

[33] Hsieh HC, Hsieh YH, Huang YH, Shen FC, Tsai HN (2005). HHR23A, a human homolog of Saccharomyces cerevisiae Rad23, regulates xeroderma pigmentosum C protein and is required for nucleotide excision repair.. Biochem Biophys Res Commun.

[34] Ng JM, Vermeulen W, van der Horst GT, Bergink S, Sugasawa K (2003). A novel regulation mechanism of DNA repair by damage-induced and RAD23-dependent stabilization of xeroderma pigmentosum group C protein.. Genes Dev.

[35] Madura K, Prakash S (1990). Transcript levels of the Saccharomyes cerevisiae DNA repair gene RAD23 increase in response to UV light and in meiosis but remain constant in the mitotic cell cycle.. Nucleic Acids Res.

[36] Reisman D, Rotter V (1993). The helix-loop-helix containing transcription factor USF binds to and transactivates the promoter of the p53 tumor suppressor gene.. Nucleic Acids Res.

[37] Tan T, Chu G (2002). p53 Binds and activates the xeroderma pigmentosum DDB2 gene in humans but not mice.. Mol Cell Biol.

[38] Ismail PM, Lu T, Sawadogo M (1999). Loss of USF transcriptional activity in breast cancer cell lines.. Oncogene.

[39] Landa I, Ruiz-Llorente S, Montero-Conde C, Inglada-Perez L, Schiavi F (2009). The variant rs1867277 in FOXE1 gene confers thyroid cancer susceptibility through the recruitment of USF1/USF2 transcription factors.. PLoS Genet.

[40] Majumder S, Ghoshal K, Li Z, Bo Y, Jacob ST (1999). Silencing of metallothionein-I gene in mouse lymphosarcoma cells by methylation.. Oncogene.

[41] Galibert MD, Baron Y (2010). Identification of specific Protein/E-box-containing DNA complexes: lessons from the ubiquitously expressed USF transcription factors of the b-HLH-LZ super family.. Methods in Molecular Biology.

[42] Galibert MD, Miyagoe Y, Meo T (1993). E-box activator of the C4 promoter is related to but distinct from the transcription factor upstream stimulating factor.. J Immunol.

[43] Metivier R, Gallais R, Tiffoche C, Le Peron C, Jurkowska RZ (2008). Cyclical DNA methylation of a transcriptionally active promoter.. Nature.

