# Supplementary figures and images for: USF-1 Is Critical for Maintaining Genome Integrity in Response to UV-Induced DNA Photolesions
Source: PLoS Genet. 2012 Jan 26;8(1):e1002470. doi: 10.1371/journal.pgen.1002470 (PMC3266871; doi:10.1371/journal.pgen.1002470)

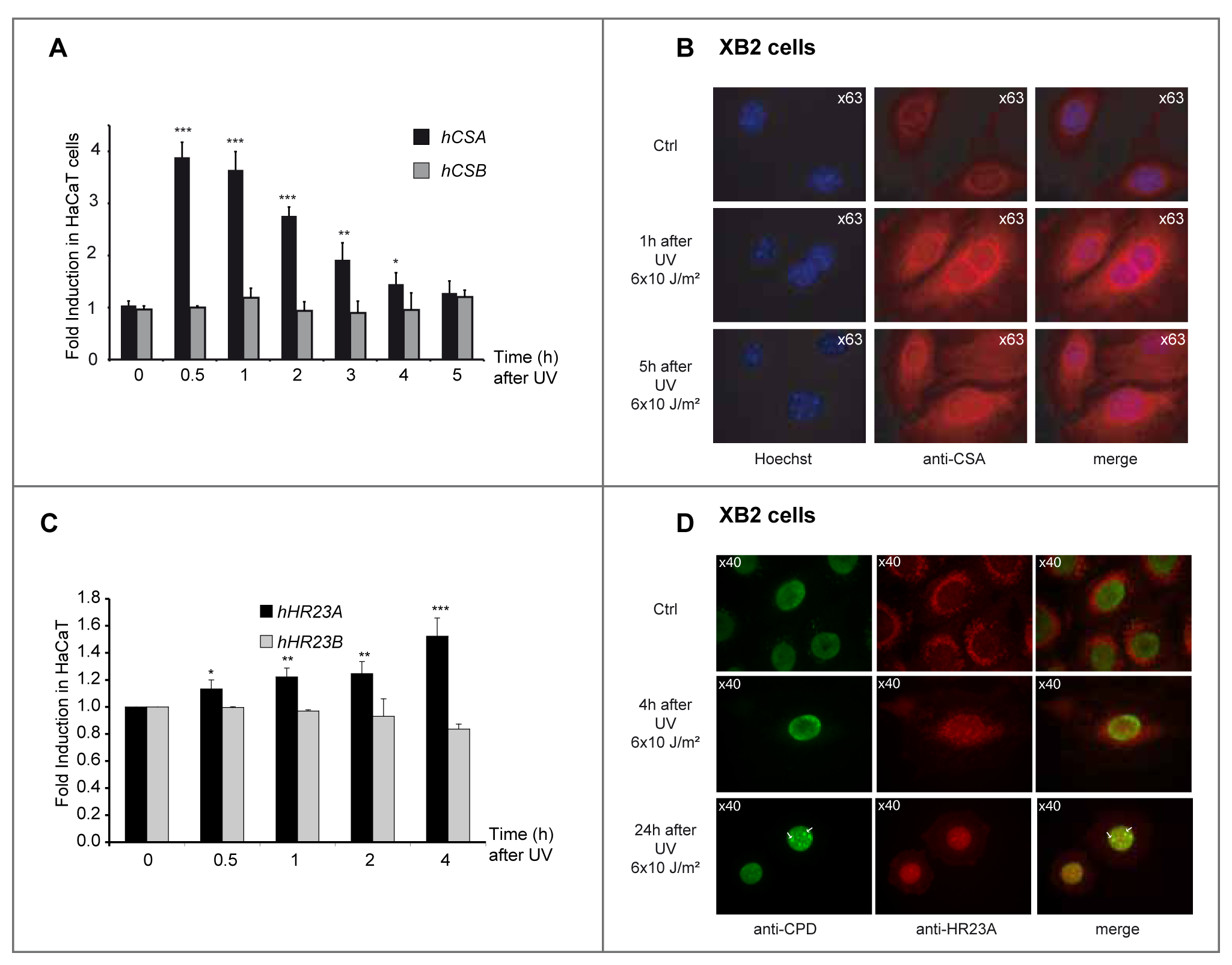

Supplement: Figure S1 — CSA and HR23A are up-regulated in p53-deficient HaCaT human keratinocytes and XB2 mice keratinocytes after UV induced DNA-damage. (A) Quantification of CSA and CSB expression in human HaCaT keratinocytes (p53 deficient cells) following UV-irradiation (8×10 J/m2) determined by RT-qPCR (ΔΔCT method). Results (n = 3) are expressed relative to control (no UV treatment) and normalized to HPRT transcript. (B) Fluoro-immunostaining microscopy (×63) performed in irradiated (6×10 J/m2) or not XB2 keratinocyte cells, and recovered 1 and 5 h post-irradiation. Detection of CSA protein was performed using the specific anti-CSA antibody (Santa Cruz) and the secondary TRITC-coupled antibody. DAPI staining was used to visualize cell nuclei. (C) Quantification of HR23A and HR23B mRNA expression in human HaCaT keratinocytes as described in (A). (D) Fluoro-immunostaining microscopy (×40) performed in XB2 keratinocyte cells, irradiated or not (6×10 J/m2), and recovered at 4 h and 24 h post-irradiation. Detection of HR23A protein was performed using the specific anti-HR23A antibody (Aviva) and the secondary TRITC-coupled antibody. Specific anti-CPD antibody (MBL) was used to visualize DNA damage (secondary antibody used was coupled to FITC). (For all results errors bars indicate s.e.m.; n = 3; one asterick, P<0.05, two asterisks, P<0.01, three asterisks, P<0.001). (TIF) [file pgen.1002470.s001.tif]
